# Editorial: Community series in inflammation in respiratory and neurological diseases and the immune-interaction of the lung-brain axis, volume II

**DOI:** 10.3389/fimmu.2025.1634921

**Published:** 2025-06-12

**Authors:** Yuyan Liu, Caicai Zhang, Wenhui Wu, Xuejing Wang, Ping Yuan

**Affiliations:** ^1^ Rehabilitation Medicine College, Shandong Second Medical University, Weifang, Shandong, China; ^2^ Department of Cardio-Pulmonary Circulation, Shanghai Pulmonary Hospital, School of Medicine,Tongji University, Shanghai, China; ^3^ Department of Physiology, Hainan Medical University, Haikou, Haikou, Hainan, China

**Keywords:** lung-brain axis, inflammation, respiratory disease, neurological disease, pulmonary hypertension

The inflammatory response has emerged as a critical determinant in the pathogenesis of a wide range of respiratory and neurological disorders, a recognition that has progressed alongside advances in immunological research. Immune cell activation and the subsequent inflammatory cascades are now widely recognized as key drivers of disease progression in both systems. Notably, pulmonary inflammation extends beyond localized vascular remodeling, exerting systemic effects—particularly on the central nervous system—through complex immune-mediated mechanisms. Growing evidence supports the existence of a lung-brain axis that facilitates this crosstalk, indicating that inflammation within the respiratory tract can influence neurological function and pathology. These findings underscore the central role of inflammation not only in shaping disease trajectories in the lungs and brain but also in mediating their interconnection. A deeper understanding of these immunoregulatory mechanisms may offer valuable insights for developing targeted therapies that address both respiratory and neurological dysfunction. In this editorial, we highlight emerging insights and key advancements that are shaping this rapidly evolving field.

## Pulmonary hypertension and the immune-inflammatory response

In recent years, there has been a growing focus on the role of immunity and inflammation in the pathogenesis of pulmonary hypertension (PH) (1). This involves various immune-related changes, such as increased activation of monocytes and macrophages, infiltration of T and B cells around blood vessels, and the formation of lymphoid-like structures near pulmonary arteries. In addition, impaired regulatory T cell function, overactive dendritic cells, and neutrophil-driven inflammation further contribute to disease progression by promoting abnormal blood vessel remodeling and damaging the lung environment (2). Zhao et al. reviewed that inflammatory cells and their chemokines and cytokines (such as IL-1β and TNF-α, and IL-6) affect pulmonary vascular system. And expounded the potential relationship of vascular cells or bone morphogenetic protein receptor 2 (BMPR2) in immune regulation. Ferrian S et al. identified TIM-3^+^ T cells and IDO-1^+^ TIM-3^+^ SAMHD1^+^ DCs as novel contributors to immune dysregulation that drives the driving progression of PAH. Notably, monocyte-derived dendritic cells (mo-DCs), along with neutrophils, play a significant role in promoting vascular remodeling and endothelial dysfunction, thereby representing promising targets for therapeutic intervention (2).

DCs exacerbate disease progression through pleiotropic cytokine secretion, particularly interleukin-6 (IL-6)—a multifunctional mediator independently associated with the pathogenesis of PH (3). B cells are involved in the development of PH by producing antibodies and pro-inflammatory cytokines. These antibodies prompt immune cells to accumulate in the vessel wall, leading to vascular inflammation, fibrosis, and vascular remodeling. Regulatory T cells (Tregs) play a balancing role in maintaining immune tolerance, and in patients with systemic sclerosis-associated pulmonary arterial hypertension (SSc-PAH), loss of Tregs function exacerbates immune dysfunction and vascular injury. Endothelial cells are stimulated by the deposition of immune complexes and cytokines, and dysfunction occurs, these changes further exacerbate the development of PH.

The development of PAH in patients with autoimmune connective tissue diseases (CTDs)—termed CTD-associated pulmonary arterial hypertension (CTD-PAH)—serves as compelling clinical evidence for the central role of maladaptive immunophenotypic changes in PAH pathophysiology, particularly through dysregulated immune cell activation and sustained inflammatory cascades. van Uden D et al. reviewed that the dendritic cell (DC) compartment comprises distinct subsets under steady-state conditions: conventional DCs, plasmacytoid DCs, and specialized AXL+Siglec6+ DCs. During inflammation, monocytes differentiate into mo-DCs. DC subset dynamics critically influence autoimmune pathogenesis and likely drive pulmonary hypertension development (idiopathic PAH/CTD-PAH) through T-cell activation and pathogenic B-cell antibody production (4).

Immune-related biomarkers have also demonstrated predictive value in PH. In chronic thromboembolic pulmonary hypertension (CTEPH), serum levels of asialoglycoprotein receptor 2 (ASGR2) correlate significantly with various immune cell parameters, particularly in relation to balloon pulmonary angioplasty (BPA). Patients with CTEPH exhibited significantly elevated ASGR2 levels prior to BPA, which decreased notably following the procedure (Xu et al.).

Beyond PH, immune dysregulation is also evident in other pulmonary conditions such as community-acquired pneumonia (CAP). Qin et al. analyzed CAP patients using machine learning and identified three immunophenotypes. Among these, Type C is significantly associated with a more severe inflammatory state and poor prognosis.

## Immune-mediated mechanisms in the fibrotic process

Immune cells and mediators are key drivers of fibrosis during tissue injury and repair, with macrophages playing a central role in both initiation and progression. Macrophage-myofibroblast transformation (MMT) has become a Research Topic in a variety of fibrosis diseases. Li et al. reviewed that macrophages are mainly activated through the TGF-β mediated Smad3 signaling pathway, which drives their differentiation to myofibroblasts expressing α-smooth muscle actin (α-SMA) and synthesizing extracellular matrix (ECM) components such as collagen. In addition to transforming growth factor (TGF-β), Notch signaling pathway and Wnt/β-catenin signaling pathway also play important regulatory roles in the MMT process (5). Fibroblasts, once seen as passive structural cells, are now recognized as immune sentinels that actively shape inflammatory responses (6). These insights highlight the dynamic crosstalk between immunity and fibrosis.

## The lung-brain axis: bidirectional interaction of immune responses between the respiratory and nervous systems

Under normal physiological conditions, the lungs and brain communicate via complex signaling pathways that help maintain systemic homeostasis. However, lung infections can disrupt this balance, leading to functional or structural changes in the brain. COVID-19 infection increases the risk of optic nerve and visual pathway disorders, potentially through viral neuroinvasion, heightened inflammation, and immune overactivation (Cao et al.).

Air pollution has emerged as a global public health concern, with growing evidence linking respiratory exposure to neurobehavioral impairments. This suggests that the lung–brain axis may be a critical conduit by which microbiome dysbiosis and environmental factors affect brain health (7). Notably, Threatt et al. reported that organic dust exposure not only intensified pulmonary inflammation but also induced neuroinflammatory responses in the murine brain.

## Targeting immune-inflammatory regulation

Zhang et al. demonstrated that *Acanthopanax senticosus* can significantly improve cognitive deficits in Alzheimer’s disease mice by promoting phosphorylation of mitogen-activated protein kinase (MAPK) signaling pathway and inhibiting the production of inflammatory factors. ATP/GTP-binding protein like 4 gene (AGBL4) promotes malignant progression of glioblastoma (GBM) by regulating matrix metalloproteinase-1 (MMP-1). Zhang et al. demonstrated that knockdown of AGBL4 inhibited the proliferation, migration, and invasive ability of GBM cells, while overexpression had the opposite effect.

Inflammation also plays a critical role in pulmonary vascular remodeling in PH (8). Li et al. highlighted the therapeutic potential of immunosuppression in treating CTD-PAH by dampening immune dysregulation. Immunosuppression strategies have reversed PH in preclinical models, suggesting their value as adjunctive treatments.

**Figure 1:**
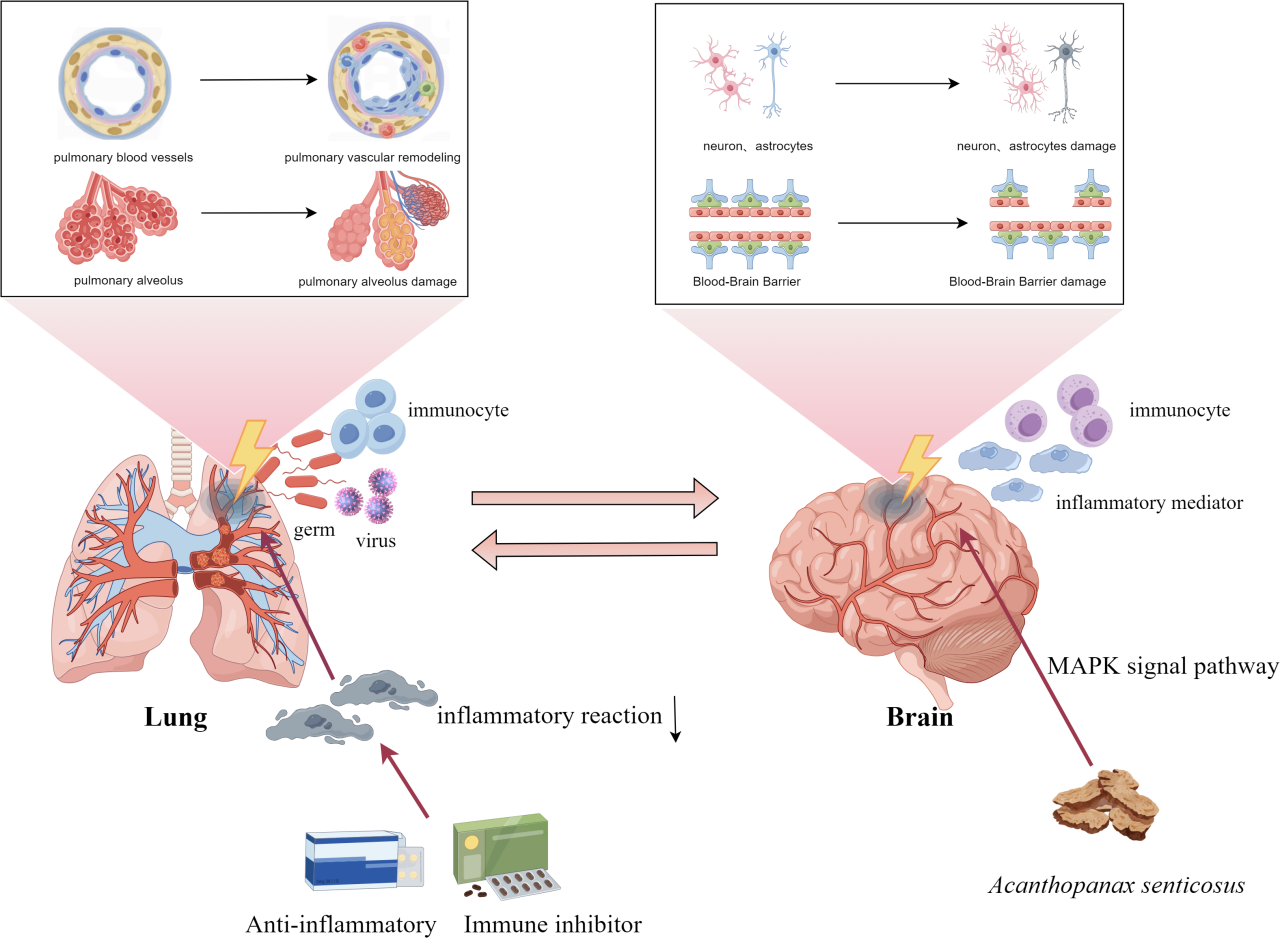
Lung-brain axis and immune response.

In summary, this study underscores the immune-inflammatory connection between respiratory and neurological diseases, highlighting shared mechanisms involving immune cells, cytokines, and signaling pathways—particularly those mediated by the lung—brain axis (**Figure 1**). While current findings point toward the promise of immunosuppression therapy, challenges remain. Future efforts should aim to precisely target immune pathways to enhance treatment specificity, and improve clinical safety.
